# The Prevalence of Low Energy Availability in Cross-Country Skiers during the Annual Cycle

**DOI:** 10.3390/nu16142279

**Published:** 2024-07-15

**Authors:** Ekaterina A. Bushmanova, Aleksandra Y. Lyudinina, Evgeny R. Bojko

**Affiliations:** Department of Ecological and Medical Physiology, Ural Branch, Institute of Physiology, Russian Academy of Sciences, Pervomaiskaya av.50, Syktyvkar 167982, Russia; salu_06@inbox.ru (A.Y.L.); boiko60@inbox.ru (E.R.B.)

**Keywords:** energy expenditure, energy intake, energy availability, fats and carbohydrates contribution, cross-country skiers

## Abstract

Background and Objectives: A sustained mismatch between energy intake (EI) and exercise energy expenditure (EEE) can lead to Low Energy Availability (LEA), as well as health and performance impairments characteristic of Relative Energy Deficiency in Sport (RED-S). Research in females has identified specific LEA cut-points for the risks of developing physiological and performance disturbances. Cut-points in males have yet to be evaluated; therefore, this study examined the prevalence of LEA in highly trained male cross-country skiers. The key purpose of this study was to analyze EI, resting energy expenditure (REE), EEE, and energy availability (EA) in highly trained cross-country skiers during the preparation and competition periods. The secondary objective of our study was to evaluate the relative contribution of fats and carbohydrates to EI, REE, and EEE. Materials and Methods: EI was determined by an estimated 24 h diet recall method, REE was assessed by indirect calorimetry, and EEE was estimated from heart rate in 27 cross-country skiers. Results: EI amounted to 4050 ± 797 kcal/day on a typical training day (TD) and 5986 ± 924 kcal/day (*p* < 0.001) on a typical competition day (CD). REE on TDs (2111 ± 294 kcal/day or 30 ± 6 kcal/day/kg) was higher (*p* < 0.05) than on CDs (1891 ± 504 kcal/day or 27 ± 7 kcal/day/kg). The EA in the athletes was <15 kcal∙kg FFM^–1^·d^–1^ on TDs and <65 kcal∙kg FFM^–1^·d^–1^ on CDs. EI was not optimal, as indicated by low EA throughout TDs (June). This could be associated with insufficient EI along with a high amount of EEE (3690.7 ± 485.2 kcal/day). During the transition from TD to CD, an increase in the contribution of fats to EI and EEE was observed in cross-country skiers. Conclusion: The conception of LEA and REDs and their potential implication for performance is underestimated among coaches and athletes. The importance of appropriate dietary strategies is essential to ensure that enough calories are consumed to support efficient training.

## 1. Introduction

The significant amount of exercise in cross-country skiing training and competitions requires coordinating energy intake (EI) and total energy expenditure (TEE) to maintain high performance across the different periods of the annual cycle. Athletes who fail to consume enough calories have an increased risk of Relative Energy Deficiency in Sport (RED-S). This syndrome affects performance and health, resulting in endocrine alterations, suppression of the reproductive axis, mental disorders, thyroid suppression, altered metabolic responses, loss of fat-free mass (FFM), impaired bone restructuring, gastrointestinal tract dysfunctions, and cardiovascular system dysfunctions and overtraining. All those consequences are a result of low energy availability (LEA). Energy availability (EA) is defined as the amount of energy that is available for physiologic function and daily activity. It is calculated by excluding the exercise energy expenditure (EEE) from EI [1,2,3,4,5,6].

Normally, cross-country skiers’ EI varies depending on the period of the annual cycle [4]. This is because, in most cases, an athlete’s actual EI does not match its expected EI [7]. Therefore, it is important to adjust EI at different stages of the training cycle according to nutritional guidelines developed for athletes [8,9,10,11]. Generally, the REE and TEE of athletes are well studied [4,6,12,13]. Most of the research is devoted to the analysis of the relationship between the EEE and body composition [4]. Studies involving a combined analysis of EI and TEE of cross-country skiers over the annual cycle are rare [14]. There are also few studies of EE and TEE in cross-country skiers during the only training period of the annual cycle [12,15]. No research has been performed on the LEA in cross-country skiers. Also, the contribution of energy substrates such as fats and carbohydrates (CHOs) to the energy supply at REE and EEE has not yet been established among cross-country skiers. A similar study of the contribution of substrates to energy expenditure during high-intensity intermittent exercise was conducted only in male cyclists [16].

In view of the above, the primary aim of our study was to analyze EI, REE, EEE, and EA in highly trained cross-country skiers across the preparation and competition periods. The secondary aim of the study was to evaluate the contribution of fats and CHOs to EI, REE, and EEE.

## 2. Methods

### 2.1. Study Design

**Figure 1:**
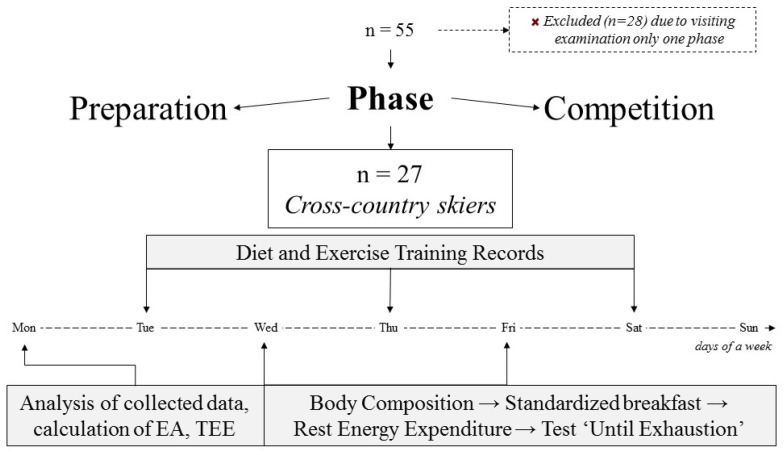
Study Design.

The prospective study on athletes’ energy availability was conducted on different days of the annual cycle: training (TD) and competition (CD). The highly trained cross-country skiers (male, *n* = 55) were involved in the experiment. We excluded 28 athletes from the experimental group due to not passing the criteria for participation (Figure 1).

Selected participants (*n* = 27) visited the laboratory on TDs and CDs to provide information on their diet and exercise training records. Body composition and REE, calculated EA and TEE, the contribution of CHOs and fats in EI, and energy expenditure were measured.

### 2.2. Participants

The current members of the national cross-country skiing team were examined on the preparation (June) and competition (March) days. The inclusion criteria were active participation in championships, sports experience (at least 5 years), age (18–30 years), absence of acute, chronic diseases (no bronchial asthma), absence of medical drug usage and alcohol intake, and mandatory two-stage examination in both periods. All cross-country skiers were under the supervision of their coaches and were following sport-specific training regimens unique to their sport.

All participants provided written informed consent regarding the study design and the risks of the experimental procedures. Some of the risks included dizziness and mild nausea during blood collection by a trained practitioner. During the test ‘Until Exhaustion’, there may be changes in blood pressure, electrocardiogram parameters, and general well-being in response to physical exertion. If significant symptoms develop, testing will be stopped. This study was approved by the Local Research Bioethics Committee of the Institute of Physiology of the Komi Scientific Centre of the Ural Branch of the Russian Academy of Sciences (approval date: 1 November 2013 and 28 December 2022) in accordance with the Declaration of Helsinki [17].

### 2.3. Procedures

#### 2.3.1. Anthropometric and Body Composition Measurements

The subjects came to the laboratory after an overnight fast (12 h fast), refraining from vigorous exercise for at least 15 h, avoiding caffeine and alcohol during the preceding 24 h, and consuming a normal evening meal the night before. Height, body mass, and body composition were estimated via the electrical impedance body composition analyzer ACCUNIQ BC380 (SELVAS Healthcare, Daejeon, Republic of Korea) in a standing upright position, bare feet, as indicated in the manual.

#### 2.3.2. Actual Dietary Intake and Exercise Training Records

All participants were informed about how to properly keep records of the three-day diet (one weekend day and two weekdays) and exercise training, which were later used to calculate the EI and EEE. Directions for measuring and recording food, along with guidance on portion size, were provided, along with the diet record forms to complete.

Participants were asked to record everything they consumed during three days during a typical training and competition period using a food and training diary. The athletes were asked to provide as many details as possible, including the brand names of the food and drinks (including performance drinks), cooking methods, and the amount of food consumed. To quantify the portion of the foods and fluids, athletes refer to the weight and volume provided on food packages or using standardized household measures consumed. They were also asked to label meals as breakfast, lunch, dinner, or snacks. The athletes were asked to specify the type of day (a rest, competition, or training day) and provide the details on what activities were carried out on each day, with accurate duration (minutes) and intensity of exercise (low, moderate, or high). To improve the reliability of the results and to ensure that athletes did not miss meals, the food diaries were reviewed and re-checked with the participation of a registered dietitian who had years of experience. Food diary data were analyzed using the specially developed computer program (version 1.1.0.328, IPhys., 2014, Russia) ‘Sport: Calculation and Analysis of the Diet’ (certificate of registration No. 2014619853, 2014, Russia) [18]. The average daily amounts of total and relative proteins, fats, and CHOs were calculated. These responses were then compared with the calculated energy and macronutrient intake levels based on moderate and high activity level recommendations provided by the International Society of Sports Nutrition (ISSN) [11].

#### 2.3.3. Rest Energy Expenditure

REE was measured by indirect calorimetry using an automated system (‘Oxycon Pro’, CareFusion, Jaeger, Wuerzberg, Germany). The REE measurements were conducted in the morning (8:30–11:00 am) after standardized breakfast [19,20] before the cycling test ‘until exhaustion’ on TDs and CDs. After entering the laboratory, the participants rested supine for 10 min prior to data collection without a gas-analyze mask. REE was determined in a supine position for 30 min in a semi-darkened, quiet room (23°). The subjects were advised to stay awake and not move or talk during the measurement. REE was determined by the minimum values of VO_2_ at rest from the data provided by the ‘Oxycon Pro’ system using the last 15 min of the data (the steady state).

#### 2.3.4. Test ‘Until Exhaustion’

The protocol included one minute of cycling without load (for adaptation) and then a stepwise load increase of 40 W in 2-minute increments starting with an initial load of 120 W. We continuously recorded heart rate, workload, and energy expenditure (every 15 s). We performed maximal oxygen uptake (VO_2_max) testing via an ergometer bicycle (Ergoselect-100, Ergoline GmbH, Bitz, Germany). The pedaling speed was 60 rpm. In ‘breath-by-breath’, VO_2_, VCO_2_, and energy expenditure were measured throughout the exercise period using an automated system (‘Oxycon Pro’, CareFusion, Jaeger, Wuerzberg, Germany).

#### 2.3.5. Energy Availability

EEE was estimated from heart rate using wearable heart rate monitors during all exercise sessions (Polar V800, Polar Electro, Kempele, Finland). EA was estimated as EI minus EEE and expressed in kcal∙kg FFM^–1^·day (d^–1^), then compared with cut-off values for low (<30 kcal∙kg FFM^–1^·d^–1^) and optimal EA (>45 kcal∙kg FFM^–1^·d^–1^) [2].

#### 2.3.6. Percentage Contribution of Macronutrients

CHO and fat contributions to EI were calculated considering that 1 g protein = 4.1 kcal, 1 g of CHO = 4.1 kcal, and 1 g fat = 9.3 kcal. From EI, the total protein intake (in kcal) was deducted, and the percentage was determined:

% Fats = (Fats, kcal·day^−1^/EI, kcal·day^−1^) × 100;
% CHOs = (CHOs, kcal·day^−1^/EI, kcal·day^−1^) × 100.

CHO and fat contributions to REE were calculated using the formula [21]:

% Fats = ((1 − RER)/0.29) × 100;
% CHOs = ((RER − 0.71)/0.29) × 100.

CHO and fat contributions to EEE were determined using a specially developed computer program, ‘Assessment of energy expenditure and the contribution of macronutrients to performance in the ‘until exhaustion’ test on the ‘Oxycon Pro’ (certificate of registration No. 2022613491, 2022, Russia).

#### 2.3.7. Energy Balance

The general EB equation is EB (kcal/day) = EI (kcal/day) − TEE (kcal/day). If EB is negative (negative EB), then TEE is larger in magnitude than EI. Likewise, if EB is positive (positive EB), then TEE is smaller in magnitude than EI.

#### 2.3.8. Total Energy Expenditure

TEE of the participants was calculated by prediction equations using the physical activity level coefficient (PAL), which is FAO/WHO/UNU recommended [22]: TEE (kcal/day) = PAL × REE (kcal/day). The PAL met the recommendations for adult elite endurance athletes based on the volume and intensity of exercises on the TD and CD [22].

#### 2.3.9. Blood Sampling

To rule out negative nitrogen balance, an assessment of the biochemical status of athletes was performed. Venous blood samples were collected from athletes after at least 12 h of fasting. The samples were collected into vacutainer tubes (Bekton Dickinson BP, Franklin Lakes, NJ, USA) containing heparin as an anticoagulant. The concentration of urea was determined by enzyme immunoassays using a biochemical analyzer ‘ChemWell 2900′ (Awareness Technology, Inc., Palm City, FL, USA).

#### 2.3.10. Statistical Analysis

All data were analyzed using the Statistica software (version 12.6, StatSoft Inc., 2015, Tulsa, OK, USA). Categorical variables are displayed as numbers and percentages, and numeric variables are presented as mean (M) and standard deviation (SD). The distribution of all numeric variables was checked using Shapiro–Wilk’s test. Non-parametric Friedman’s analysis of variance was used to compare groups. Spearman’s rank correlation coefficient (Rs) was calculated to determine relations between variables. The significance level was set at *p*-values < 0.05.

## 3. Results

A summary of the physical and anthropometric characteristics of participants is presented in Table 1.

### 3.1. Actual Nutrient Intakes

The EI (kcal/day) of cross-country skiers was lower by 10% of the recommended calorie intake [11] on TDs and was higher by 10% of the recommended calorie intake on CDs. Total CHO and protein intake were below the recommended values, and total fat intake, in contrast, was higher in both periods. Detailed information is provided in Table 2.

Total CHOs (*p* < 0.001), fats (*p* < 0.001), and protein intake (*p* = 0.266) on CDs were higher than on TDs in absolute (g/day) and relative (g/day/kg) values. The expected direct correlations between VO_2_max and EEE in the test ‘until exhaustion’ (Rs = 0.76; *p* < 0.001) were revealed. The direct correlations between VO_2_max and total CHO intake (Rs = 0.35; *p* = 0.009) were also demonstrated. The positive correlation between the test time and total fat (Rs = 0.31; *p* = 0.022), CHO (Rs = 0.30; *p* = 0.024), and protein (Rs = 0.31; *p* = 0.019) intake was discovered.

### 3.2. Energy Expenditure, Energy Availability

EA and its specific components (EI, EEE, and FFM) were measured, and energy expenditure for the total sample group can be found in Table 3.

Athletes had LEA on TDs, whereas EA was optimal for athletes on CDs. A positive correlation between REE (kcal/day), percentage fat mass (Rs = 0.60; *p* < 0.001), and FFM (Rs = 0.51; *p* < 0.01) was found on CDs. The predicted TEE on TDs amounted to 5067.6 ± 706.0 kcal, which was significantly higher than that on CDs—4537.9 ± 1209.1 kcal (*p* < 0.001).

When comparing daily EI and predicted TEE, a calculated relative energy deficit of ~1000 kcal was found during high-intensive TDs, which was not observed on CDs. Moreover, urea in venous blood was 6.9 mmol/L (with limits of 3.9–12.6 mmol/L) on TDs and 4.8 mmol/L (with limits of 2.8–6.7 mmol/L) on CDs, which was within the reference ranges of urea (1.7–8.3 mmol/L), indicating the absence of negative nitrogen balance in athletes.

### 3.3. Fats and Carbohydrates Contribution

**Figure 2:**
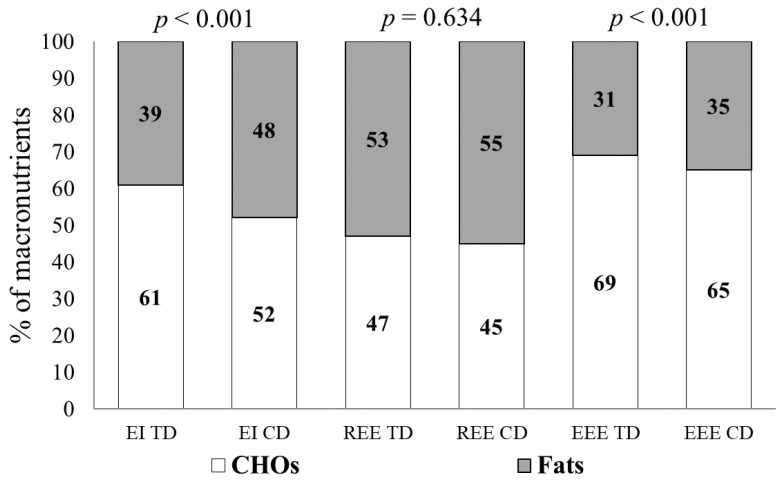
Percentage contribution of carbohydrates and fats to energy intake and energy expenditure. Note: TD—training day; CD—competition day; EI—energy intake; REE—resting energy expenditure; EEE—exercise energy expenditure; CHOs—carbohydrates.

The percentage of fats to CHOs in EI, REE, and EEE structure is presented in Figure 2.

The percentage ratio of the fat and CHO contribution to the athletes’ diet structure during TDs was 39% to 61%, and on CDs, it was 48% to 52% (*p* < 0.001). The ratio of fats and CHOs in REE during TDs was 53% to 47%, and on CDs, it was 55% to 45% (*p* = 0.634), respectively. The ratio of fats and CHOs in the EEE was 31% to 69% during TDs and 35% to 65% (*p* < 0.001) on CDs. From TD to CT, an increase in the fats contribution to EI and EEE in cross-country skiers’ was observed.

## 4. Discussion

In our research, we found high variability in EI (from 2289 to 6538 kcal) in cross-country skiers (Table 2), which was consistent with the literature data [16,18,19]. Only 44% of cross-country skiers under study followed the recommended standard in diet for endurance athletes during TDs, while other athletes’ diets had energy values below 4000 kcal on TDs. On CDs, however, all athletes followed the dietary guidelines. Analysis of EI in cross-country skiers showed lower CHO contribution in EI from TD to CD. In contrast, fats contribution was higher than recommended for endurance athletes, which could lead to induction of fatigue, overtraining, and reduced immune resistance [16,18]. In research [23], biochemical indicators showed the presence of overtraining in hockey players whose fat intake levels exceeded the norms. It is worth noting that the similarities between the symptoms of overtraining and RED-S are significant. Both have a hypothalamic–pituitary origin and can be influenced by low CHOs and LEA [24].

Endurance athletes should follow the diet that includes 144 g/day of protein, 100 g/day of fats, and 541 g/day of CHOs on TDs with moderate intensity exercise, and the diet that includes 162 g/day of protein, 120 g/day of fats, and 722 g/day of CHO intake on CDs with high-intensity exercise [11]. The observed increased fat intake likely compensated for the total energy deficit in our athletes’ diets. In fact, during the survey, almost all respondents noted that they had to satisfy their food needs on their own by buying extra groceries at stores (dairy products, fruits, nuts, and sweets, or fast food), a practice which is not uncommon among athletes [25] on TDs and especially on CDs.

The VO_2_max and time in the test ‘until exhaustion’ are high-performance indicators for cross-country skiers. The direct correlation between VO_2_max and macronutrient consumption confirmed their explicit participation in the energy supply of EEE.

In fact, REE is the largest TEE component; therefore, the measurement and interpretation of REE is an important part of the effective training process [6]. In our study, we found no difference between the absolute REE values (kcal/day) when transitioning from TD to CD, while there was a reliable difference (*p* = 0.035) between the relative REE values (per kg weight), suggesting that the relative REE values are more informative than the absolute REE values. We established correlations between REE and FFM, as well as between REE and fat mass (%) on CDs. We suppose that the REE level is directly dependent on these parameters.

Additionally, the difference in REE values could be regarded as an effect of regular training or as an outcome of changed diets [13]. The analysis in the review [1] led us to the conclusion that intense training experienced by athletes on a TD increases REE significantly. It was anticipated that cross-country skiers with a minimum of five years of experience in the sport would demonstrate increased REE. Aerobic exercise also increases REE, but this effect may depend on its mode and intensity, as well as on an athlete’s fitness level [26]. Since more than 80% of training time on a TD is associated with aerobic exercise, we can assume that higher REE values are caused to a greater extent by adaptation to exercise.

A non-significant REE decrease on CDs could be due to a reduction of training load compared with TDs. We know that the competitive activity of athletes places them in stressful situations, which are accompanied by increased activity of the endocrine system, leading to active mobilization of energy substrates. In addition to these factors, EEE can influence thyroid status, protein metabolism, circulating leptin, thermogenesis, stimulation of *β*-adrenergic functions, and mitochondrial liver activity [3,7].

The majority of EA research has been conducted in female populations. As RED-S gains attention in the scientific community, more research is now addressing male athletes, the need to determine the severity of this issue, and how best to prevent health and performance detriments. However, the number of male-based studies is limited, and for that reason, findings from highly trained male cross-country skiers are addressed here. The studies indicate that athletes experience considerable energy deficits on certain preparation or competition days [3,27], which is a consequence of LEA [1]. The athletes may also unintentionally run into LEA during periods with increased training volume or when engaging in sports with high energy expenditure (e.g., cross-country skiing) as well as at low EI [1,4,7,9]. In our study, during TDs of high-intensity exercise, EA values were <15 kcal∙kg FFM^–1^·d^–1^, which was in accordance with the studies showing the prevalence of LEA in athletes [2,6]. Collectively, these studies [1,4,7,9] indicate a prevalence of greater than 50% of athletes who are at risk for LEA, which agrees with the current study. This finding indicates a need to educate athletes about EA and the importance of maintaining adequate levels of actual nutrient intake.

The energy value of an athlete’s diet corresponding to TEE is one of the most important conditions for an effective training process. In our study, the predicted TEE values in cross-country skiers on TDs were higher than those on CDs, which implies that skiing is an energy-intensive sport. On high-intensity TDs and during ski racing competitions, these values can be approximately 4800–6000 kcal/day [10], sometimes reaching 8000 kcal. During the CDs, they may exceed 10,000 kcal [19].

The preparation phase of the annual cycle is characterized by predominantly high-volume training at moderate intensities, which improves endurance capacity and provides more efficient use of fuel substrates. During the later part of the preparation phase, training volume is reduced while intensity is gradually increased. The goal of this phase is to reach peak performance and to transfer the training effects into the competition phase, where exercise intensity is the highest. In the week before an important competition, volume and intensity are typically decreased to allow the body to optimally recover for competition [28]. In our study, however, we observed the opposite. In some intensive days of the preparation phase, the body of an athlete is exposed to a prolonged state of LEA, which depletes the body’s functional reserves. Therefore, when approaching important competitions, many athletes might not achieve their maximal results due to a depleted energy reserve. The most obvious explanation for these energy deficits was likely the classical issue of under-reporting EI through self-assessment in athletes. Such under-reporting can amount to 10–45% of the TEE [29]. Another explanation for why the athletes showed energy deficits could emerge from a low accuracy of the 24-hour recall method used to estimate EI in athletes. Finally, LEA during TDs could be explained by the fact that CHO and protein intake were reduced in 78% and in 89% of the athletes, respectively, relative to the normative values for high-intensity exercise during TDs. The reference level of urea across TDs and CDs indicates the absence of negative nitrogen balance in athletes.

According to the EB concept, EB is defined as the state achieved when EI equals TEE. The literature analysis showed that most athletes of cyclic sports exhibited obvious negative EB values, both on certain days of preparation and in the competition period [3]. Negative EB on TDs has also been noted among male and female cross-country skiers [14,15], male runners [27,30], and female lightweight rowers [31]. The energy deficits averaged 304 kcal/day (4.7% of the TEE) in men. Positive EB on the TDs was identified in male cross-country skiers [15]. On the CDs, negative EB was recorded in male cross-country skiers [14], male cyclists [32,33,34,35], and male and female runners [35]. The energy deficit averaged 2177 kcal/day in men and 1252 kcal/day in women. Positive EB on the CDs was found in swimmers, both male and female [36]. On TDs, a small energy deficit leading to increased use of energy reserves may be desirable for coaches and athletes to achieve maximum fitness, but in the competition period, the energy balance must be maintained at proper levels [1]. A study was carried out simulating ‘the Tour de France race’ in the metabolic chamber to calculate the daily EB based on the values of TEE and EI [37]. A positive daily EB was found on active recovery days, while a significantly negative daily EB was observed on exercise days.

Among the factors affecting the achievement of high results in cross-country skiing, great importance is given to the exceptional work of the oxygen transport system and the ability of skeletal muscles to oxidize fats and CHOs, which are of great significance in the supply of energy to ensure physical [1] and athletic aerobic performance [38]. The highest priority factors in the distribution of energy substrates during exercise are the load duration [39] and intensity [40]. Intramuscular triglycerides and glycogen do not contribute significantly to energy production under exercise intensity, amounting to 25% VO_2_max. Under loads of submaximal intensity (40–65% VO_2_max), there is an equal maximum rate of fat oxidation and high availability of free fatty acids (FAs) due to lipolysis in peripheral and intramuscular adipocytes [40]. A progressive decline in the metabolism of FAs, with exercise intensity increasing from 65 to 85% VO_2_max, is apparently compensated for by a progressive increase in blood glucose turnover. Thus, the contribution of plasma substrates to calorie expenditure remains constant over a wide range of exercise intensities (i.e., 25–85% VO_2_max) [40].

During training, fats are oxidized in low- and moderate-intensity exercise, while CHOs are oxidized mainly in high-intensity exercise; the contribution of fat oxidation to TEE during exercise with a VO_2_max above 85% is usually ignored [41]. Our earlier studies of highly trained cross-country skiers at rest during the general training period and study of physical activity in the test ‘until exhaustion’ and competition (1.3 and 15 km races) showed identically modified serum-saturated FA profiles (especially medium-chain FAs). These modifications manifested as considerable increases in the concentrations of medium-chain FAs (capric, lauric, and myristic), with no changes in long-chain fatty acid concentrations relative to baseline levels [42]. Another study indicated that the dietary levels of *α*-linolenic acid should be increased in athletes because of the associated increase in the body’s aerobic potential via the use of essential FAs [38]. In this work (Figure 2), we found a decrease in the CHO (%) contribution and a corresponding increase in the proportion of fats (%) in EEE as the intensity of the load increased, which was fully consistent with the results of previous studies [16]. The percentage difference in evaluated energy expenditure confirmed that athletes effectively utilize fats at high intensities when transitioning to CDs. It should be noted (Table 1) that athletes had a significantly lower fat mass in the competition period than in the training period (9% and 11%, respectively), although both parameters displayed were within the reference for cross-country skiers. It can be assumed that a significant increase in the contribution of fats to EEE when transitioning to CDs is attributed to an elevated rate of fat utilization that coincides with an increased VO2max, which is reflected in the research [41].

Although the upper limit of lipolysis in humans is unknown, fat mobilization and oxidation were more intense in athletes with high aerobic performance [42]. Moreover, in studies using indirect calorimetry, when the intensity of exercise increased, the anaerobic energy system was activated, and additional CO_2_ was produced due to the activation of the bicarbonate buffer system, a process that can cause overestimation of the share of CHOs and underestimation of the share of fats [41]; hence, further study of the degree of participation of macronutrients in exercise of different intensity levels is deemed relevant.

## 5. Limitations

This study is not without limitations. Future research should focus on the use of more accurate dietary intake and energy expenditure data collection techniques. As a prevalence study, the present sample size was relatively small. The demands of a 3-day diet and training records caused a substantial participant burden. Another limitation of this study was the self-reported nature of all the data and the potential recall bias. Furthermore, there remains no standard for determining EI. In the field (on CDs), assessment or screening of EI and energy expenditure are time-consuming and also include a degree of error. On CDs, athletes experience emotional stress. In this regard, not all data obtained in laboratory conditions can be unconditionally projected to sporting competitions. In addition, when working with athletes, there is always the possibility of individual differences and deviations. TEE was estimated with an equation rather than determined with the reference method (i.e., doubly-labeled water), which probably led to an overestimation of TEE in this study.

## 6. Conclusions

The presented study showed that at the beginning of the training period (June), the highly trained cross-country skiers had a nutritional imbalance that could affect their performance during the competition period. By the time of the State Cross-Country Ski Championship (March), the EI was 10% higher than the recommended levels, predominantly because of the excessive fat intake. The absolute values of REE (kcal/day) did not differ between the annual cycle periods, but at the same time, the relative REE values (per kg of body mass) decreased significantly on the CDs in comparison with the TDs. Lower EEE values on the CDs correlated directly with the changes in the body composition, namely with the FFM and with the fat percentage, which likely affected the decrease of the predicted TEE on the CDs. The EA levels in cross-country skiers were classified as low on certain days of the training period and as optimal during the competition period. In the structure of the REE and EEE, we observed the increased contribution of fats correlating with the decreased contribution of CHOs when the athletes transitioned from the TD to the CD. Thus, the LEA in cross-country skiers observed at the beginning of the TD was probably caused by insufficient EI, increased REE, and EEE during the high-intensity and high-volume training loads in the days we examined the skiers.

## Figures and Tables

**Table 1:** Summary of the characteristics of the participants.

Characteristics	TD	CD
Age, years	21.1 ± 3.9	21.1 ± 4.0
Body height, cm	178.3 ± 5.7	178.4 ± 5.3
Body mass, kg	73.3 ± 4.3	70.1 ± 4.8
Body mass index, kg/m^2^	22.4 ± 1.3	22.0 ± 1.4
Fat mass, %	10.7 ± 3.3	8.9 ± 2.9 *
VO_2_max, mL·min^−1^·kg^−1^	59.7 ± 6.9	62.2 ± 10.2 *

Note: M ± SD, *—*p* < 0.05 denotes a significant main effect between TD and CD. VO_2_max—maximal oxygen consumption; TD—training day; CD—competition day.

**Table 2:** Actual nutrient intakes in highly trained cross-country skiers (male).

Period		Total PRO Intake	Total Fats Intake	Total CHOs Intake	EI, kcal/Day
TD	Actual intake (g/day)	129.7 ± 33.7	147.0 ± 35.5	516.6 ± 123.4	4050.2 ± 796.6
	Actual intake (g/kg/day)	1.9 ± 0.5	2.1 ± 0.5	4.4 ± 1.7	54.6 ± 10.6
	Recommended intake (g/day)	144.3 ± 33.8	100.2 ± 23.5	541.3 ± 126.8	4511 ± 1056
CD	Actual intake (g/day)	150.4 ± 17.5	276.7 ± 51.5 ***	666.7 ± 122.5 ***	5986.3 ± 923.7 **
	Actual intake (g/kg/day)	2.1 ± 0.3	3.8 ± 0.9 ***	9.2 ± 2.0 ***	79.2 ± 16.2 **
	Recommended intake (g/day)	162.4 ± 38.0	120.3 ± 28.2	721.7 ± 169.0	5413 ± 1268

Note: M ± SD, ***—*p* < 0.001, **—*p* < 0.01 denotes significant main effect between TD and CD. TD—training day; CD—competition day; CHOs—carbohydrates; PRO—proteins; EI—energy intake.

**Table 3:** Energy availability calculation variables and components in highly trained cross-country skiers (male).

Variables	TD	CD
Energy Availability, kcal∙kg FFM^–1^·d^–1^	14.8 ± 9.3	64.4 ± 4.5 **
Energy Intake, kcal/day	4050.2 ± 796.6	5986.3 ± 923.7 **
Exercise Energy Expenditure, kcal/day	3690.7 ± 485.2	777.4 ± 67.8 **
Rest Energy Expenditure, kcal/day	2111.2 ± 294.3	1891.6 ± 504.9 **
Rest Energy Expenditure, kcal/day/kg	30.4 ± 5.6	27.0 ± 6.8 **
Fat-free mass, kg	65.7 ± 9.9	63.7 ± 4.2

Note: M ± SD, **—*p* < 0.01 denotes a significant main effect between TD and CD. TD—training day; CD—competition day.

## Data Availability

Data are all contained within the article.

## Abbreviations

CD	competition day
CHOs	carbohydrates
EA	energy availability
EB	energy balance
EEE	exercise energy expenditure
EI	energy intake
FAs	fatty acids
FFM	fat-free mass
PAL	physical activity level
REE	rest energy expenditure
TD	training day
TEE	total energy expenditure
VO_2_max	maximal oxygen uptake
